# Laboratory growth of denitrifying water column microbial consortia from deep-sea shipwrecks in the northern Gulf of Mexico

**DOI:** 10.12688/f1000research.12713.3

**Published:** 2018-08-06

**Authors:** Dhanya Haridas, Justin C. Biffinger, Thomas J. Boyd, Preston A. Fulmer, Leila J. Hamdan, Lisa A. Fitzgerald

**Affiliations:** 1 American Society for Engineering Education, Washington, DC, USA; 2 Chemistry Department, University of Dayton, Dayton, OH, USA; 3 Naval Research Laboratory, Washington, DC, USA; 4 University of Southern Mississippi, Hattiesburg, MS, USA

**Keywords:** shipwreck, denitrification, microbe, 16S, Gulf of Mexico

## Abstract

**Background**: Shipwrecks serve as a rich source for novel microbial populations that have largely remained undiscovered. Low temperatures, lack of sunlight, and the availability of substrates derived from the shipwreck’s hull and cargo may provide an environment in which microbes can develop unique metabolic adaptations.

**Methods**: To test our hypothesis that shipwrecks could influence the microbial population involved in denitrification when a consortium is grown in the laboratory, we collected samples proximate to two steel shipwrecks in the northern Gulf of Mexico. Then under laboratory conditions, we grew two independent denitrifying microbial consortia. Each consortium was grown by using the BART assay system and analyzed based on growth kinetics, ion chromatography and 16S amplicon sequencing.

**Results**: Both denitrifying consortia were different from each other based on varied growth profiles, rates of nitrate utilization and 16S amplicon sequencing.

**Conclusions**: Our observations conclude that the laboratory grown water column microbial consortia from deep-sea shipwrecks in the Gulf of Mexico are able to undergo aggressive denitrification.

## Introduction

The biogeochemical process that transforms dissolved inorganic nitrogen to nitrogen gas is known as denitrification (DN). This metabolic pathway impacts the nitrogen (N) cycle by returning elemental N to the atmosphere ^1, 2^. It can alternatively be defined as the reduction of more oxidized forms of nitrogen (NO _3_^-^, NO _2_^-^, NO and N _2_O) to N _2_ gas, where it can be linked to the oxidation of iron, sulfur and reduced carbon species ^3^. It is primarily performed by facultative heterotrophic or chemolithoautotrophic bacteria under anoxic or very low-oxygen conditions ^3^, where microorganisms utilize nitrate or nitrite as the terminal electron acceptor ^4^. DN, alongside other biogeochemical processes (carbon and sulfur cycles), plays a key role in maintaining the nutrient balance in marine habitats ^5^.

In recent years, shipwrecks have been identified as areas from which novel microbial species have been isolated, because of the introduction of foreign material to the area ^6^. Thus, they would be an ideal location to discover unique microorganisms and metabolic activity, as these areas are known to be diverse habitats for macroorganisms in the marine environment ^7^. The goal of this research was to prospect for novel DN microbial consortia near deep-sea shipwrecks in the Gulf of Mexico, culture the consortia under laboratory conditions and determine their DN activity. In this study, we collected water samples proximal to two steel shipwreck sites located in the northern part of the Gulf of Mexico, and analyzed the denitrifying and culturing potential of the microbial consortia obtained from the two sites.

## Methods

### Environmental sampling

We obtained water samples ~600 m down current from two steel-hulled shipwrecks investigated as part of the Shipwreck Corrosion, Hydrocarbon Exposure, Microbiology and Archaeology (SCHEMA) study, which addresses the effect of the 2010 *Deepwater Horizon* spill on deep-sea shipwrecks in the northern Gulf of Mexico ( http://www.boem.gov/GOM-SCHEMA/). Samples were collected onboard the R/V *Pelican* using a CTD-rosette during the PE15-22 expedition in May 2015. The shipwreck *Halo,* is a steel-hulled steam tanker, resting in ~140 m of water, and ~50 miles west of the Mississippi River’s Southwest Pass. The double steel-hulled German U-Boat *U-166* shipwreck rests in ~1400 m of water within 10 km of the Macondo wellhead, the epicenter of the 2010 *Deepwater Horizon* spill. The water samples were stored in sterile plastic bottles at 4°C until further use.

### Enrichment of denitrifying microbial consortium

The commercially available denitrifying Biological Activity Reaction Test (DN-BART) assay (HACH, Colorado, USA) was used to enrich for DN bacteria. Briefly, the lyophilized media in the DN-BART was solubilized with 15 mL water sample from either the *Halo* or *U-166* shipwreck site. The assay was performed based on manufacturer’s instructions, with the exception that the assay was incubated for 30 days instead of the suggested 4 days. The enriched microbial consortium obtained from the DN-BART assay was used as the inoculum to perform the growth curve and Ion Chromatographic (IC) studies.

### Growth curve studies

Nunc tubes (Chemglass, NJ, USA) containing 10 mL of modified Indole Nitrite medium (pancreatic digest of casein 20 g/L, disodium phosphate 2 g/L, dextrose 1 g/L, potassium nitrate 1 g/L) were used for all assays. Sterile nitrogen gas was bubbled through the media for 15 min prior to inoculation to de-gas and establish an anaerobic environment. The enriched *Halo* and *U-166* DN consortium derived from the DN-BART assay was used as the inoculum to perform the growth curve studies. Each tube was inoculated with 100 µL of DN-BART consortium. The inoculated Nunc tubes were analyzed for a period of 24 h at 30°C (Excella E25, Fisher Scientific, MA, USA). The optical density of the samples was measured at 600 nm (OD _600_) every 2 h post-inoculation using the Spectronic 200 Spectrophotometer (Thermo Scientific, PA, USA) over a 24 h period after inoculation. All inoculated samples were done in triplicates.

### Ion chromatography studies

The nitrate/nitrite concentrations were tracked by IC using a Dionex IC-3000 IC fitted with an IonPac AS16 column. The mobile phase was 9 mM Na _2_CO _3_ at 1.0 mL/min and a Dionex 7 anion standard mix was used for calibration before and half way through sample runs. The DN microbial consortium was cultured similarly to the growth curve assay ( *i.e.* anaerobically to enable the denitrifying conditions to develop). The *Halo* and *U-166* cultures were sampled (1 mL) using a sterile syringe and needle (BD, NJ, USA) every 2 h over a period of 24 h from the inoculated Nunc tubes. The sample was centrifuged at 12,000 × *g* for 3 min in a sterile 1.5 mL eppendorf tube (Eppendorf, NY, USA). The supernatant was additionally syringe filtered (0.2 µm filter Syringefilter, SC, USA) and stored in 1 mL IC vials (Thermo Scientific, Pittsburg, PA). All analyzes were performed in duplicate or triplicate. Nitrate concentrations were often above the calibration level (100 mg/L) and are annotated as estimated values ( *J*). Standard errors for replicate measurements ranged from 0 to 2.26% with an average of 0.31% for the aggregate runs.

### 16S amplicon sequencing

To determine the DN phylotypes present in the DN consortia, genomic DNA was isolated using DNA isolation solutions I, II and III (bioWORLD, Dublin, OH) from the *Halo* and *U-166* denitrifying microbial consortia after 24 h of growth and 16S amplicon sequencing of the V4 region and bioinformatics analysis was performed by Seqmatic (Fremont, CA). High throughput NGS was performed using the Illumina MiSeq platform using 2x250bp reads and the FASTQ data was processed using the Qiime pipeline.

## Results

### Hydrographic conditions of the water samples

The depth, temperature, salinity and dissolved oxygen (DO) of the water samples obtained from the region proximal to the *Halo* and *U-166* shipwreck sites were obtained with the CTD ( Table 1). The water sample obtained proximal to the *U-166* shipwreck site had a higher DO content (6.6 mg/L) and lower temperature (4.3°C), as compared to the *Halo* shipwreck site that had 4.1 mg/L DO and 17.7°C. This indicates that both the water samples have varied hydrographic conditions.

**Table 1.** Hydrographic conditions of water column samples collected proximate to two steel shipwrecks *Halo* and *U-166* in the northern part of Gulf of Mexico. The depth, temperature, salinity and dissolved oxygen (DO) for the water samples collected from *Halo* and *U-166* are listed below.

	*Halo*	*U-166*
Depth (m)	141.3	1448.9
Temp (°C)	17.7	4.3
Salinity (PSU)	36.3	35.0
DO (mg/L)	4.1	6.6

### Denitrifying microbial consortia

Both water samples were also analyzed for the presence of denitrifying microbial consortia using the commercially available DN-BART assay. Upon performing the assay, it was observed that *Halo* and *U-166* water samples did not produce foam or bubbles around the ball or in the tube after 4 days, the recommended duration for developing a positive reaction. Hence, due to the nature of the unique water samples, the assay was continued for 30 days. Following the 30 days, foam was detected around the ball, providing evidence for potential DN microbial consortia from the *Halo* and *U-166* shipwreck sites. The DN consortia that were enriched using the DN-BART assay were further analyzed for microbial growth, nitrate/nitrite media concentrations and microbial composition over 24 h.

### Growth profile of the DN microbial consortium

**Figure 1.**
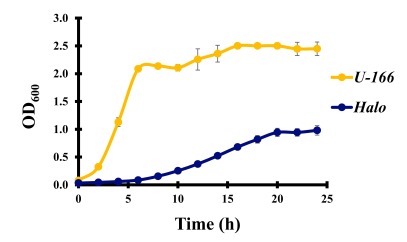
Growth curves from *Halo* and *U-166* denitrifying population. The growth profiles of both DN consortia were analyzed for a period of 24 h at 0D _600_. The *U-166* DN consortia grew at a much faster rate compared to the *Halo* DN consortia.

To determine the growth profile of the DN consortia, the growth curve assay was performed. It was observed that the DN consortia from *Halo* grew (OD _600_ = 0.980) much slower than the *U-166* consortia (OD _600_ = 2.448) over the 24 h period ( Figure 1; Dataset 1). Thus providing the first evidence that both the DN consortia are different from each other. All analyses were performed in triplicate ( Figure 1).

### Nitrate and nitrite ion chromatography analysis

**Figure 2.**
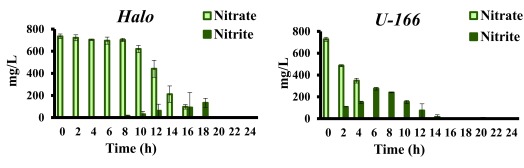
Ion chromatographic studies of the DN microbial consortium isolated from *Halo* and *U-166* shipwreck sites. Samples were collected every 2 h for a period of 24 h and nitrate and nitrite levels were determined. Note: Nitrate values were above the calibration level (100 mg/L) and are thus estimates (but proportional).

Ion chromatography (IC) studies were performed to identify the denitrifying potential of the isolated microbial consortia. The *Halo* microbial supernatants showed a steady decline in nitrate concentration (734 mg/L to 0.7 mg/L) as microbial growth entered into the logarithmic growth phase. As the nitrate concentration decreased, there was an increase in nitrite concentration from 1.4 mg/L, to a maximum of 130 mg/L and tapered down to 4.3 mg/L at 24 h. The *U-166* microbial consortium rapidly converted nitrate into nitrite, as shown with a decrease in nitrate concentration (730 mg/L to 2.5 mg/L) followed by an increase in nitrite concentration (0 to 240 mg/L), which was later followed by a subsequent decrease in nitrite levels to 2.2 mg/L ( Figure 2; Dataset 2).

### 16S amplicon sequencing

**Figure 3.**
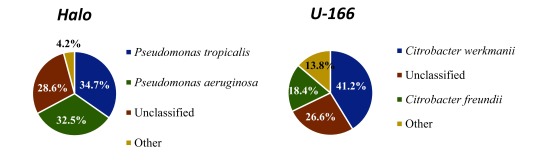
16S amplicon sequencing on the *Halo* and *U-166* DN consortium after 24 h culturing in Indole Nitrite medium. Genomic DNA was isolated from the *Halo* and the *U-166* DN consortium and the V4 region of the 16S was analyzed.

Since the growth curve and IC studies indicated that the DN consortia from *Halo* and *U-166* are mutually exclusive, we wanted to determine the microbial composition of both *Halo* and *U-166* DN consortia using 16S amplicon sequencing. The *Halo* DN consortium primarily consisted of the *Pseudomonas* genus (98.1%), while the *U-166* DN consortium was dominated by the *Citrobacter* genus (72.6%). At the species level, *P. tropicalis* and *P. aeruginosa* for *Halo,* and *C. werkmanii* and *C. freundii* for *U-166* were primarily detected ( Figure 3; Dataset 3)), thus indicating that both DN consortia are mutually exclusive.

Growth curve assay: Growth curve studies were performed over a 24 h period for both the Halo and U-166 DN consortia The optical density of the cultures were measured every 2 h at 600nm. Click here for additional data file. Copyright: © 2018 Haridas D et al. 2018 Data associated with the article are available under the terms of the Creative Commons Zero "No rights reserved" data waiver (CC0 1.0 Public domain dedication).

Ion chromatography studies: Nitrate and nitrite levels of the DN consortia isolated from Halo and U-166 sites were determined every 2 h for a period of 24 h using ion chromatography Click here for additional data file. Copyright: © 2018 Haridas D et al. 2018 Data associated with the article are available under the terms of the Creative Commons Zero "No rights reserved" data waiver (CC0 1.0 Public domain dedication).

16S amplicon sequencing: Genomic DNA was isolated from DN consortia after 24 h of growth and the V4 region was analyzed using the 16S metagenomics sequencing Click here for additional data file. Copyright: © 2018 Haridas D et al. 2018 Data associated with the article are available under the terms of the Creative Commons Zero "No rights reserved" data waiver (CC0 1.0 Public domain dedication).

## Discussion

The deep sea, identified with shelf depths greater than 200 m, has been documented to be the largest hypoxic and anoxic environment present on earth ^8^. The varied living conditions mentioned earlier induces microbes to adopt unique metabolic adaptations. Hence, the marine dark biosphere has been recognized as a rich resource of unique microbial populations. Apart from the unique microbial life detected in the marine dark biosphere, shipwreck sites located in the deep sea also serve as a rich source of distinct flora and fauna ^6^. Using two different shipwrecks at varying depth and material allows for the comparison of the metabolic activity of DN microbial consortia isolated from steel shipwreck sites.

One of the biggest challenges in characterizing new microbes from the deep-sea is the ability to successfully culture them in the laboratory. The initial approach to identifying a DN consortium was to assess growth using the commercially available DN-BART assay. The DN-BART assay provided the necessary nutrients in a modified nitrate medium and the presence of a potential DN microbial consortium from both *Halo* and *U-166* shipwreck sites was confirmed. To further characterize the DN microbial consortia, the turbidity of the media was monitored and the nitrate/nitrite concentrations were examined every 2 h over a 24 h time period.

The water sample from the *Halo* shipwreck site was able to grow under the conditions set forth in this study, but at a much slower rate when compared to *U-166*. When the *Halo* microbial consortium began its logarithmic growth, there was a steady decline in the nitrate concentration and a subsequent increase in the nitrite concentration in the supernatant. The *U-166* DN consortium also grew and the turbidity of the culture was greater as compared to *Halo* DN consortium. The IC studies further corroborated the decrease in nitrate levels and a concurrent increase in the nitrite concentration during the logarithmic phase of growth. Further, the *U-166* consortia consumed nitrite, most likely as nitrate was completely consumed, at a rate 2 times slower than that observed in the *Halo* DN consortium (30 mg/L compared to 65 mg/L respectively). To determine the DN phylotypes present in the DN consortia, 16S amplicon sequencing was performed, and it was observed that at the species level *P. tropicalis* and *P. aeruginosa* for *Halo* and *C. werkmanii* and *C. freundii* for *U-166* were the most dominant and are known denitrifiers ^9, 10^. It was also observed that the *Citrobacter* dominating the *U-166* DN consortia consumed nitrate at a rate that was faster than other industrial microbial consortia containing *Citrobacter* adapted for denitrification ^11^.

This study indicates that *Halo* and *U-166* were good prospecting sites for novel microbial consortia related to denitrification. The water mass passing by each shipwreck site has a distinct DN consortium which can be grown under laboratory settings. The *U-166* DN microbial consortium performs denitrification at a much faster rate than the *Halo* DN microbial consortium and most known industrial microbial consortia. This elevated DN activity could be the result of local hydrodynamic conditions or the proximity to the shipwreck, but additional studies are needed to identify the exact parameters. In conclusion, both DN consortia isolated from novel prospecting sites (shipwrecks) in the Gulf of Mexico can be cultured in the laboratory and can utilize a DN metabolic pathway for growth.

## Data availability

The data referenced by this article are under copyright with the following copyright statement: Copyright: © 2018 Haridas D et al.

Data associated with the article are available under the terms of the Creative Commons Zero "No rights reserved" data waiver (CC0 1.0 Public domain dedication). 

**Dataset 1:** Growth curve assay: Growth curve studies were performed over a 24 h period for both the *Halo* and *U-166* DN consortia. The optical density of the cultures were measured every 2 h at 600nm. DOI, 10.5256/f1000research.12713.d179765 ^12^

**Dataset 2:** Ion chromatography studies: Nitrate and nitrite levels of the DN consortia isolated from *Halo* and *U-166* sites were determined every 2 h for a period of 24 h using ion chromatography. DOI, 10.5256/f1000research.12713.d179766 ^13^

**Dataset 3:** 16S amplicon sequencing: Genomic DNA was isolated from DN consortia after 24 h of growth and the V4 region was analyzed using the 16S metagenomics sequencing. DOI, 10.5256/f1000research.12713.d179767 ^14^
