# Supplementary material for: Laboratory growth of denitrifying water column microbial consortia from deep-sea shipwrecks in the northern Gulf of Mexico
Source: F1000Res. 2018 Aug 6;6:1834. Originally published 2017 Oct 13. [Version 3] doi: 10.12688/f1000research.12713.3 (PMC6069730; doi:10.12688/f1000research.12713.3)
Supplement: Peer review history

# Peer review: Referee response for version 3

**DOI:** 10.5256/f1000research.17320.r36863

**Published:** 2018-08-09

**Authors:** Dimitri Kalenitchenko

**Affiliations:** 1 Université Laval, Quebec City, QC, Canada

The manuscript version that has been submitted answered all the comments made on previous version.

I have read this submission. I believe that I have an appropriate level of expertise to confirm that it is of an acceptable scientific standard.

# Peer review: Referee response for version 2

**DOI:** 10.5256/f1000research.16633.r34921

**Published:** 2018-07-27

**Authors:** Dimitri Kalenitchenko

**Affiliations:** 1 Université Laval, Quebec City, QC, Canada

The revised version of "Laboratory growth of denitrifying water column microbial consortia from deep-sea shipwrecks in the northern Gulf of Mexico", allows a better interpretation of the data thanks to the new mat and met section. I still have concern about the fact that the method used by the author can't really test that each shipwreck has a particular DN consortium. I can understand that samples are difficult to obtain but I would recommend to change a little bit the last paragraph to temper your words.

 For example instead of saying "Each shipwreck site" say "The Water mass passing by each shipwreck site". It is a small difference but I think a reader that only read the last section of the discussion would think that the shipwreck itself has its own DN consortium. It might be true but it is not what you test for.

I have read this submission. I believe that I have an appropriate level of expertise to confirm that it is of an acceptable scientific standard.

# Response

**Published:** 2018-08-03

**Authors:** Dhanya Haridas

**Affiliations:** US Naval Research Laboratory, USA

Dear Reviewer,

 Thank you for taking the time and reviewing the paper again. We have included the edit suggested by you in the discussion.

 Respectfully,

 Dhanya Haridas

# Peer review: Referee response for version 1

**DOI:** 10.5256/f1000research.13771.r31968

**Published:** 2018-03-29

**Authors:** Dimitri Kalenitchenko

**Affiliations:** 1 Université Laval, Quebec City, QC, Canada

The paper entitled “Laboratory growth of denitrifying water column microbial consortia from deep-sea shipwrecks in the northern Gulf of Mexico” by Haridas *et al*. explores the potential of steel shipwrecks to host a specialized community with a focus on denitrifying bacteria. Overall the paper is well written and easy to follow, their results are relevant but before suggesting the acceptance of the manuscript, I suggest that authors resubmit a modified version of the manuscript that include a detailed material and method section.

 16S amplicon sequencing section :

- Please provide the DNA extraction kit details
- Please provide the name of the sequencing platform and primers
- Please explain the bioinformatics treatment including the taxonomic affiliation used

 

 Denitrifying microbial consortia :

- Why did the authors extend the incubation time up to 30 days, I am wondering if they checked for contamination from external source.

16S amplicon sequencing :

- I am very surprised about the very low diversity they obtained, I agree that they enriched the community in DN bacteria but with only two tubes for two conditions I am not convinced they do not just select for a contaminant or a bacterium in each tube.
- How did the authors obtained these percentages, BLAST ?

 

 Discussion

 I think the conclusion  that shipwreck are good prospecting area for novel microbial consortia is highly speculative based on these data especially without a reference point before the water mass flow across the wreck.

I have read this submission. I believe that I have an appropriate level of expertise to confirm that it is of an acceptable scientific standard, however I have significant reservations, as outlined above.

# Response

**Published:** 2018-06-06

**Authors:** Dhanya Haridas

**Affiliations:** US Naval Research Laboratory, USA

Dear Reviewer,

Thank you for taking the time to review and suggest comments/edits to the manuscript. We have included information about the DNA isolation kit, the sequencing platform on which the 16S metagenomics studies were performed and the bioinformatics pipeline used. We hope the reviewer will find the responses satisfactory.

You expressed your concern on the need for us to extend the incubation time from 4 days to 30 days for the BART assay. We appreciate the reviewer raising this concern and the reason we decided to extend the incubation time was due to the uncharacterized marine environmental samples used and it is known the concentration of viable microbes is less (as determined by a 3H-Leucine incorporation assay) than when grown in a rich medium. Hence extending the incubation time in this study resulted in an increased concentration of the viable microbial culture which helped determine if the assay was positive for microbes which could perform denitrification.

You mentioned another concern about the study selecting for a contaminant. The raw data (excel file) containing all the sequencing tags obtained from the 16S metagenomics sequencing has been provided. The authors would like to mention that the microbial population obtained from the BART assay served as the starting inoculum for all the assays mentioned in the paper and therefore any potential aerobic or facultative aerobic bacteria cultured in the BART assay medium would not have propagated in the Nunc tube due to the anaerobic nature of the environment, thus further diminishing the diversity and potential for an aerobic contaminant. The percentages were part of the bioinformatics report submitted to us by Seqmatic. For example, in the Halo sample the total number of reads at the species level was 169,918 of which 59,117 reads were classified as *Pseudomonas tropicalis* (By Seqmatic) thus making it 34.72% of the total species population.

We hope we addressed all your concerns in this response and thank you once again for reviewing the manuscript. 

Respectively,

Dhanya Haridas

# Peer review: Referee response for version 1

**DOI:** 10.5256/f1000research.13771.r31967

**Published:** 2018-03-26

**Authors:** Maxim Ru­bin Blum

**Affiliations:** 1 Department of Symbiosis, Max Planck Institute for Marine Microbiology , Bremen, Germany; 2 Israel Oceanographic and Limnological Research, Haifa, Israel

This study aims to examine the cultivable denitrifying microbial consortia from marine environment near deep-sea shipwrecks in the Gulf of Mexico. The authors hypothesized that the shipwrecks could influence the microbial population involved in denitrification. The methods used in this study, however, have not adequately tested this hypothesis, as no control samples were obtained. On the other hand, the authors were able to report enrichment of two interesting bacterial consortia, one of which appeared to perform denitrification at a much faster rate than that measured for most known industrial microbial consortia, providing an opportunity to study these bacteria in more detail in the future.  

 If I understood correctly, only a single water sample from each location was used for the denitrifying Biological Activity Reaction Test, allowing to isolate a denitrifying consortium per sampling site. It is likely that these bacteria occur in a patchy manner in the environment, thus further sampling may reveal very different denitrifying consortia. Broadening the sampling effort is needed to test if either environmental factors or presence of shipwrecks affects the diversity of denitrifying bacteria.

I have read this submission. I believe that I have an appropriate level of expertise to confirm that it is of an acceptable scientific standard.
